# Human immunity

**DOI:** 10.70962/jhi.20250001

**Published:** 2025-02-26

**Authors:** Jean-Laurent Casanova

**Affiliations:** 1 St. Giles Laboratory of Human Genetics of Infectious Diseases, The Rockefeller University, New York, NY, USA; 2 Howard Hughes Medical Institute, New York, NY, USA; 3 Laboratory of Human Genetics of Infectious Diseases, INSERM, Necker Hospital for Sick Children, Paris, France; 4 Imagine Institute, Paris Cité University, Paris, France; 5 Department of Pediatrics, Necker Hospital for Sick Children, Paris, France

## Abstract

Due to the burden of infectious diseases, human life expectancy at birth remained about 20–25 years until the end of the 19th century, implying that host defense—which operates at the individual level, and only poorly at that—is barely sufficient at population level. Microbes preceded us by three billion years and evolve much more rapidly. Moreover, protective immunity has been selected at the evolutionary cost of allergy, autoinflammation, and autoimmunity. It is therefore no exaggeration to predict that almost all humans carry inborn errors of immunity, with insufficient or excessive responses to some environmental triggers, infectious or otherwise. Thanks to the remarkable power of its concepts and recent progress in its methods, genetics has finally made it possible to investigate the mechanisms of human immunity at the molecular and cellular levels. Human inborn errors provide countless opportunities to analyze immunity and its derailments in natural conditions, at an unprecedented scale, and are thus a unique asset from both biological and medical perspectives. Hence, the *Journal of Human Immunity*.

## Teaser

Human immunity is often insufficient against infectious agents and sometimes over-reactive to innocuous components of the environment or the human body. Genetic, immunological, and clinical studies of inborn errors of immunity—where responses are either too weak or too strong—offer unprecedented insights into the physiology and pathology of host defense. This is the focus of the *Journal of Human Immunity*.

## Introduction

I am both thrilled and daunted by the prospect of writing this inaugural editorial of the *Journal of Human Immunity*. I am sure that it has not escaped the notice of readers that the title of this new journal is similar in structure to those of the other three major journals published by the Rockefeller University Press: the *Journal of Experimental Medicine*, the *Journal of General Physiology*, and the *Journal of Cell Biology*. Stepping into the shoes of three giants—William Welch in 1896, Jacques Loeb in 1918, and George E. Palade in 1955—in 2025 is irresistibly exciting and terrifying.

A new scientific journal is needed when a new field of study matures to such a point that it can no longer be considered adequately or sufficiently covered by existing journals. In other words, a new journal is needed when the scientists in the field concerned feel the need to have their own venue for publication. This time typically coincides with the moment at which a scientific community becomes aware that its growth and success depend on it having a medium for communication not only within, but also beyond, the field. With hindsight, given the spectacular medical and biological contributions of human inborn errors of immunity since the 1950s, this launch of the *Journal of Human Immunity* is long overdue.

## Human inborn errors

As a tribute to Archibald Garrod, monogenic disorders in this field are referred to as “inborn errors,” despite the old-fashioned nature of this term. They can also be described as conditions due to single-gene mutations or variants. With his “inborn errors of metabolism,” Garrod is arguably the founder of human genetics, and certainly the founder of its Mendelian branch (1). As an introduction to the workings of the extraordinary mind of Garrod, I recommend that my trainees and younger colleagues read the biography written by Alexander G. Bearn, an eminent and erudite physician-scientist who was on the faculty at Rockefeller before becoming a trustee (2).

George Beadle, who, together with Edward Tatum, developed the one gene–one enzyme paradigm, famously asserted that they merely rediscovered in molds what Garrod had discovered four decades earlier in humans (3). He rightly saw Garrod as the founder of biochemical genetics and stressed that Garrod, like Mendel and his peas before him, was way ahead of his time. As we all know, European scholars remained ignorant of Mendel’s breakthrough for four whole decades.

Paradoxically, the Mendelian school of genetics, with its simple solutions to complicated problems, has always been less popular than the biometrician school and its more complicated solutions. More than a few geneticists interested in physiology or medicine have preferred to follow the approaches of Francis Galton and Karl Pearson, inspired by the brilliant mathematical equations of these scientists despite their uncertain physiological relevance.

In some circles, a prejudice against inborn errors still persists in 2025. The human genetics landscape today is reminiscent of that at the turn of the 20th century; the rift between Mendelians and biometricians has not fully healed. Despite the avalanche of sensational achievements by molecular Mendelians, from Linus Pauling and Vernon Ingram’s discovery of the molecular basis of sickle cell disease onward (4, 5), some modern biometricians still do not fully appreciate the importance of inborn errors.

Inseparable from Mendel’s notion of monogenic inheritance lies Garrod’s seminal but insufficiently appreciated notion of “chemical individuality” (1). It implies that causality and the mechanism of human health and disease operate at the individual, as opposed to population, level. This profound idea is consistent with Ernst Mayr’s “population thinking,” through which he stressed that populations were exceedingly heterogeneous (6). Inspired by the concepts of Mendel and Garrod, studies of human inborn errors may stand the test of time. We should be proud of our heritage and try to maintain this illustrious tradition.

## Inborn errors of immunity

In the field of inborn errors of immunity, as in other fields, facts have much more impact than words, and the extraordinary success of this field already attests to its viability and vitality. At least 600 human inborn errors of immunity have been characterized at both the molecular genotypic and clinical phenotypic levels (7, 8). The rate of discovery of single-gene lesions affecting immunity has grown exponentially since 1985, when Stuart Orkin discovered the molecular basis of autosomal recessive adenosine deaminase deficiency (9).

The transition from classical to molecular genetics made it possible to characterize biochemically complete or partial defects underlying autosomal or X-linked recessive disorders (10, 11, 12) and even pseudoautosomal recessive disorders (13). It also revealed the existence of semi-dominant inborn errors and led to the classification of dominant disorders into three major groups—operating via haplo-insufficiency, negative dominance, or gain of function—or, more rarely, by the creation of a novel function (14), or by the separation of functions (15), reminding us that genes can be pleiotropic. Finally, the high degree of genetic and allelic heterogeneity underlying almost any clinical or immunological phenotype was soon found to be matched by the unsuspected wide range of phenotypes resulting from different genotypes at almost any given locus (16, 17).

Inborn errors of immunity were initially thought to be Mendelian traits with complete clinical penetrance, but molecular genetic studies have revealed that many, probably even most, display incomplete penetrance and thus should be seen as “monogenic but not Mendelian.” Clarifying the mechanism of incomplete penetrance holds the tantalizing hope of molecularly mapping the uncharted regions of “non-Mendelian genetics.” Approaches benefiting from both a solid base camp (a causal monogenic lesion) and a set compass (an immunological mechanism of disease) have the edge over purely mathematical approaches (18, 19, 20, 21).

Now, inborn errors of immunity are probably more often sporadic than familial due to incomplete penetrance, the occurrence of conditions caused by de novo mutations and the decline in sibship size worldwide. There has been a gradual transition from studies of multiplex to sporadic kindreds. Moreover, forward genetics approaches in patients with no immunological phenotype have identified surprising causal genes and mechanisms of disease (10, 11). These discoveries have also led to a gradual shift of the field from immunologically to clinically defined conditions.

The alleles underlying the three known examples of monogenic resistance to infection are common due to pathogen-driven natural selection, but it was traditionally thought that inborn errors of immunity were necessarily due to rare alleles, especially for those underlying infections (22). Surprisingly, some have turned out to be due to common alleles. This is illustrated not only by *MEFV* variants common in the Mediterranean basin that underlie familial Mediterranean fever in a semi-dominant manner (23, 24), but also by a *TYK2* variant common in Europeans, in whom it underlies a recessive susceptibility to tuberculosis (25), and even *IFNAR1*- and *IFNAR2*-null variants common in the Pacific and Arctic regions, respectively, and underlying viral diseases in a recessive manner (26, 27, 28).

Finally, the field of human inborn errors of immunity has expanded with the description of “clinical phenocopies,” such as those driven by somatic genetic variants (29), and autoantibodies neutralizing components of host defense (30). Admittedly, these autoantibodies may be genetically driven, in which case they are not phenocopies in the strict sense of the term (31). They could then be seen as consequences of a distinctive set of inborn errors of tolerance to self. Moreover, some somatic deficits do not even have a known germline counterpart (32), and germline and somatic lesions can act in concert (33).

## Biological and medical breakthroughs

Work in this field has demonstrated that human infection, inflammation, virus-induced cancer, autoimmunity, and allergy can be genetic. Examples include pneumococcal disease in patients with agammaglobulinemia (34) and papillomavirus-induced skin cancer in humans with epidermodysplasia verruciformis (35, 36). The impact of the field on the study of immunity to infection has been remarkable, with the discovery that genetic heterogeneity underlies physiological homogeneity in almost every infection studied (37, 38). Evidence that autoimmunity can be genetic was also provided by the discovery that deficits of certain complement components underlie systemic lupus erythematosus (39, 40).

The concept of autoinflammation was born in this field with the descriptions of Mediterranean fever and Aicardi-Goutières syndrome (41, 42). Although not strictly speaking, allergic, hereditary angioedema paved the way for a series of elegant molecular studies on allergy (43, 44). Within each of these five categories—human infection, inflammation, virus-induced cancer, autoimmunity, and allergy—genotypic studies have revealed a much wider range of clinical phenotypes than was initially suspected (45). We may now even wonder whether there is any severe clinical phenotype that cannot, in principle, be caused by an inborn error of immunity (46).

Listing all the molecular triumphs of the field would require a book (47), but we can cite a few breakthroughs here (10, 11). New gene products of the utmost importance have been discovered, including BTK and AIRE, which play fundamental roles in B and T cell biology, respectively (48, 49, 50, 51, 52, 53). In the myeloid lineage, the important gene products discovered include CYBB and other proteins responsible for the phagocytic respiratory burst (54, 55). This field has also defined the physiological range of ubiquitously expressed type I IFNs, both genetic deficits and excesses of which are pathogenic, results reminiscent of the findings of endocrinologists in their studies of hormones (56). Finally, the first mutations of a nuclear RNA gene were found in patients with cartilage hair hypoplasia (57), whereas the first mutations affecting epigenetic processes were found in patients with immunodeficiency, centromere instability, and facial abnormalities (58).

This field has also broken new ground in the realm of treatment, including the first successful immunoglobulin treatment (59), the first successful hematopoietic stem cell transplantation (60), the second successful enzyme treatment (61), and the first evidence for beneficial somatic genetic reversion of a germline defect (62), paving the way for the first encouraging results for gene transfer (63) and gene editing therapies (64). Finally, it also provided the first population-based evidence of a mechanism and of the benefits of a newborn screening program, as children with severe combined immunodeficiency diagnosed on the basis of low circulating levels of TRECs at birth had better outcomes than those diagnosed later due to the possibility of performing hematopoietic stem cell transplantation at a younger age, enabling these children to remain free from infection (65).

Overall, countless patients worldwide have benefited from progress in this field, in which many scientific breakthroughs have been made. It is hard to think of a more successful field in biomedical research today, from both medical and biological angles, particularly in areas relating to genetics, immunity, infectious diseases, and pediatrics. The breadth and depth of this field are both unprecedented and unparalleled, with monumental advances in terms of both basic biology and public health.

Our field is still moving forward at an ever-increasing pace, with no signs of an inflexion point on the horizon. Thanks to the in-depth mechanistic nature of immunological studies, we can even rigorously incriminate lesions of known or new genes in single patients (66). Moreover, while many causal genes affect cell components that are specifically involved in host defense, often in leukocytes, other defects affecting ubiquitously expressed housekeeping genes have also been identified that underlie not only broad (67, 68), but also very narrow, clinical phenotypes (69, 70). Thus, only the tip of the iceberg has been revealed, with much left to discover.

## A need for greater financial support

The advent of molecular genetics has brought the field to a new dimension. For four decades, from the 1940s onwards, only about a dozen inborn errors of immunity were known; their definition was phenotypic and they were considered to be familial and rare (about 40 were known in 1983, including 12 disorders of complement [71]). The genotypic redefinition of inborn errors of immunity, from the 1980s onwards, revealed the existence of hundreds (nearly 600 in 2025) (7, 8) of such defects and showed that they could be sporadic or common.

Inborn errors of immunity and their phenocopies have turned out to be collectively much more common than initially thought. Some are even individually common. This notion is groundbreaking, from a public health angle, and it is regrettable that it remains under the radar of most governmental organizations. For example, autoantibodies neutralizing type I IFNs are found in about 0.5% and 5% of individuals under and over 70 years old, respectively, corresponding to about 100 million individuals worldwide (72, 73), and can account for an unprecedented proportion of cases of infectious diseases (74).

Human inborn errors of immunity and their phenocopies may be the rule, rather than the exception, in human populations (46). Probing this prediction will require not only further forward genetics studies, but also reverse genetics programs. The genomes or exomes of millions of people are now known. Most, if not all, viable knockout genotypes may be present in the seven billion people currently alive. There may even be deficits of each isoform and RNA gene.

Querying the growing number of large genetic databases corresponding to healthy and sick individuals in an agnostic manner, starting with a gene of interest, with no preconceived hypothesis about the phenotype, will be rewarding. As some genotypes may be lethal in utero, it may also be useful to assemble genetic databases for miscarriages. The field of inborn errors of fetal immunity also remains unexplored. Why do some fetuses suffer or even die from severe cytomegalovirus or zika virus infection, for example, whereas others do not?

Indeed, it is important to bear in mind that most human inborn errors of immunity are life-threatening, often affecting young patients, and that all affect global fitness. This field does not focus on the genetic basis of height, weight, or sleeping pattern. We study human death, including the patient’s death or its equivalent, an inability to reproduce, at population level. This is more than just a detail, as many scientific administrations like to restrict what they call “Mendelian genetics” (with no clear definition) to what they call “extreme phenotypes” (with no better definition).

We humbly suggest that death may be seen as an extreme phenotype, perhaps the most extreme. In this light, we see no reason for not expanding our search for “non-Mendelian monogenic death,” especially in patients who fall ill before puberty or during their reproductive years. We thus encourage governmental agencies and philanthropic foundations worldwide to consider the extraordinary achievements of the field over the last 80 years, as well as its even greater potential, in terms of both basic biology and public health, and to provide much more funding for such research than they currently do.

## From immunology to immunity

In this context, why not call this journal the *Journal of Human Inborn Errors of Immunity*? There are three reasons for not doing so, in increasing order of importance: (1) this title would be too long, even without “Human,” which cannot be deleted; (2) phenocopies of inborn errors of immunity are one of the most active areas of research in the field; and (3) we believe that human immunity generally is best studied with a knowledge of its genetic basis. We hope to convince human immunologists not familiar with genetics to rally to our field and to adopt our fertile and versatile approach to revisiting immunology. Hence, the *Journal of Human Immunity*.

So, why a journal of human *immunity* and not a journal of human *immunology*? Obviously, the aim is to be consistent with “inborn errors of immunity.” In addition, immunology spent its first 60 years as an “immunochemistry” focusing on antibodies and the next 60 years focusing on lymphocytes after becoming an “immunobiology” with the discovery of T and B cells in the 1960s. Myeloid cells were not rediscovered until later by immunologists, mostly as antigen-presenting cells rather than effector cells. As Tom Kindt and Don Capra elegantly put it, the history of immunology is essentially the pursuit of the “antibody enigma” (75); it has not been a pursuit of the “infection enigma” (37).

There is every reason to think that host defense is the task of all >500 cell types in our body, if only because a myriad of pathogens can attack them all. Fending off microbes can hardly be a mission exclusive to the leukocytes of the “immune system,” even if all microbes crossed an epithelial barrier via a purely mechanical breach. Not only are epithelial barriers involved, but so must be most, if not all, of the cells of the body, as recently illustrated by the genetic dissection of herpes simplex encephalitis, which has already identified 20 inborn errors of brain immunity (76). The production of most complement components in the liver had already been reported by the end of the 1960s (77). Observations of the production of antiviral type I interferons by fibroblasts and other non-leukocytic cells were also amply documented in the 1960s (78). Nevertheless, both complement and interferons have long remained at the margins of mainstream immunology.

Thus, we can consider any cell or molecule controlling infection as contributing to immunity. By inference, any abnormality of the processes in which these cells and molecules are involved, even in the absence of infection, may be considered a derailment of immunity. Blood- and tissue-resident leukocytes and their products remain central to host defense, operating as connectors, like neurons and vessels, or hormones and metabolites. However, all tissues contribute to protective immunity against microbes, the derailment of which, in any direction, can underlie “immunological” clinical phenotypes.

Why has the contribution of non-leukocytic cells to immunity not yet become mainstream immunological knowledge despite emerging interest in non-leukocytic “intrinsic immunity” (79, 80, 81, 82)? Scientific enterprise is a human endeavor. Scientific communities inevitably tend to become prisoners not only of their own data, but also of assumptions, interpretations, and inferences, which Thomas Kuhn collectively called “paradigms” and Michael Polanyi called “tacit knowledge” (83, 84, 85). The edifice holds until there are too many observations that do not fit, and there is then a shift of paradigm.

A good example in endocrinology was provided by Roger Guillemin, who identified hypothalamic hormones that regulate the pituitary gland (86). After years of debate, endocrinology finally extended from the classical endocrine glands to incorporate a region of the central nervous system; neuroendocrinology had a molecular basis. Whole-organism physiology can reveal surprising paradigm-shifting connections. In this context, our choice of “immunity” is a reminder that while we should not be quixotic, we should also not be prisoners of the current immunological paradigm, which remains centered on the immune system and its leukocytes.

## Historical roadblocks in human immunology

And why a journal of *human* immunity? First, because what we learn about human immunology has medical implications, directly when we study pathological problems and indirectly when we study physiological problems. Physiology and pathology are two sides of the same coin, studies in each of these fields nurturing the other. If Rockefeller’s motto, “science for the benefit of humanity,” were to be taken literally, or even only seriously, immunologists would prioritize human studies. Human immunopathology, as a discipline, necessarily relies on the incessant study of human immunological conditions, as exemplified at Rockefeller by the work of Henry Kunkel (87).

An equally important, but sometimes neglected, reason is that the study of humans taps into a unique resource of seven billion individuals continuously exposed to immensely diverse environmental challenges. Despite the considerable heterogeneity of medical care, no other species is “phenotyped” in natural conditions as closely as ours is at this gigantic scale. What is discovered in humans is therefore of enormous biological value to anyone interested in the immunological interface of host–environment interactions.

Mark Davis graciously adopted an expression we proposed in 2002: the “human model” (88, 89). Of course, as humans, we cannot see ourselves as a model organism, or the conditions that occur in humans as models of these conditions. The human “model” is therefore not really a model in the literal sense of the term, but it is so figuratively, as there is no better living species in which to study host defense and its many derailments in natural conditions (90).

Nevertheless, immunologists historically turned to animal studies, and for two good reasons: genetic and moral. One was that this made it possible to control the germline genetic basis of host responses to challenges, antigenic, infectious, or otherwise, which seemed technically impossible in humans. Studying immunity in a human population of unknown genetic structure is of course even more challenging than studying randomly selected crosses of randomly selected inbred C57BL/6, DBA/2, BALB/c, or other strains of mice.

Human immunology has always been limited by our immense interindividual genetic diversity. It is possible to test whether some features documented in an animal species are valid in humans, but they must, by definition, be common to all or most humans tested. Such features can delineate a general architecture of immunity, but their low granularity is an inherent biological and medical limitation, as the seven billion humans on Earth are all different, particularly when confronted with infectious agents and other environmental challenges, and understanding these differences is the ultimate goal of immunology.

The other major problem with human studies has been the moral barrier to experimentation. Exceptions included vaccinations and, in rarer circumstances, inoculations with live pathogens under medical surveillance. Despite the power of human-induced pluripotent stem cell–based studies in vitro (91) and creative approaches to the study of human tissues ex vivo (92), there is no doubt that a much broader range of experiments can be performed in inbred mice or other animals than in human cells or tissues (93). Both the genetic and experimental problems seemed unsurmountable. For this reason, immunologists turned to other vertebrates, and even invertebrates (94, 95). Meanwhile, of course, studies of plant immunity were following their own magnificent path (96), and evolutionary immunologists elegantly studied many other species (97), but in neither case were these organisms considered to be “models” for humans.

## On the shoulders of genetics

In this context, a small group of physician-scientists launched the field of human inborn errors of immunity. Rolf Kostmann and Ogden Bruton are widely seen as the two founders of the field, with their descriptions of autosomal recessive congenital neutropenia and X-linked recessive agammaglobulinemia in 1950 and 1952, respectively (34, 98). Wilhelm Lutz was recently recognized as another unsung hero, with his description of epidermodysplasia verruciformis, an autosomal recessive predisposition to viral skin warts, in 1946 (35).

The field rapidly made considerable contributions to immunology. The clinical and immunological differences between the forms of agammaglobulinemia described by Bruton and Hitzig played a key role in the delineation of T and B cells by Max D. Cooper and Robert A. Good in the early 1960s (99, 100, 101) but had less influence on Jacques F.A.P. Miller (102). Di George’s syndrome soon enriched the landscape (103). Other pioneers included Charles Janeway, David Gitlin, and Henry Kunkel. The rest is history (104).

The advent of human inborn errors of immunity offered the possibility of long-term solutions to the two problems of human immunology: the unknown and diverse genetic background, and the moral impossibility of performing most experiments in humans. Analyses of the impact of a single-locus genotype across many families and ethnicities offered greater genetic robustness than the study of single mutants in a single strain of inbred mice (93). A genetic lesion underlying a phenotype in patients of Inuit, Pygmy, and French descent is more robustly causal than a genetic deficiency engineered and tested in C57BL/6 mice.

Moreover, experimentation by humans was not required, as nature performs its own experiments. This concept of “experiments of nature” was pioneered by William Harvey, Thomas Addison, William Osler, Pierre Marie, and others and was related to genetics by Archibald Garrod, as beautifully reviewed by Irvine McQuarrie (105). Garrod remarkably asserted that “One of Nature’s experiments, the placing of a lesion in some particular spot, may serve to reveal the functions of the part affected” (106). Reminiscing about his own career, Robert A. Good wrote about his mentor in Minnesota that “McQuarrie’s concepts concerning the importance of Experiments of Nature shaped all of my research in immunology” (107).

Nature brings human genes and environmental triggers into confrontation in natural conditions, thereby revealing a great many phenotypes and genotypes. Known triggers include the thousands of known pathogens, the far greater number of other microbes, and countless allergens and carcinogens. Importantly, infectious agents have co-evolved with us. The species barrier, resulting from three billion years of evolution, is a fundamental concept in host–pathogen interaction and renders attempts to rely on animal species that are “permissive” to human pathogens a challenging endeavor of uncertain physiological and pathological relevance.

We do not call into question here the immense strides made with animal studies, nor their bright future, if only because of the almost unlimited range of experiments that can be performed with animals (93). That would be utterly foolish. It would be equally foolish, however, to think that human studies have not and cannot make considerable contributions. We question the idea that animal models represent the only way forward and that such studies are the alpha and omega of immunology. We think that the study of humans, rooted in genetics, is enlightening, thanks to the immense heterogeneity of hosts challenged naturally in a diversity of environmental circumstances. Our own population is a promised land for immunological expeditions.

## Human biology

Human biology neatly illustrates Krogh’s principle (108); it does not contradict it. This idea that one can find an ideal living species to tackle any biological problem was earlier proposed by Claude Bernard (109, 110). Illustrious examples include *Escherichia* for the operon, *Neurospora* for the one gene–one enzyme concept, *Drosophila* for the linkage of genes, *Caenorhabditis* for the development of neurons, and *Tetrahymena* for the histone code.

These extraordinary achievements do not imply that every biological problem must be tested in the most remote or exotic species, and particularly not in humans. Molecular, cell, and even whole-organism biology has thrived in non-human species. However, with recent progress in genetics, human studies have proved to be of added value in all branches of whole-organism biology. Sydney Brenner, whose launch of the study of *C. elegans* was met with enormous enthusiasm, unfortunately gained less traction for his late epiphany concerning the tremendous potential of human genetic studies (111, 112).

Defining what genes do in natural, as opposed to experimental, conditions through genetic studies of experiments of nature is a unique asset of human biology. Genetics is the only discipline at the intersection of the two branches of biology: physiology and evolution. The field of human inborn errors is therefore of direct evolutionary relevance. These studies, immunological or otherwise, shed light on the evolutionary forces operating here and now on human genes.

Of course, these studies do not tell us what happened in the past. As a means of gaining evolutionary insights, population genetic studies, including studies of ancient DNA, have unique added value (113). The synergy between patient-based physiological studies and population-based evolutionary studies is considerable, whereas combining patient-based evolutionary studies and population-based physiological studies does not readily rescue the intrinsic weaknesses of either approach.

Indeed, inspired by Garrod, we believe that it is better to understand one patient (almost) completely than to understand a thousand (at most) partially. Population geneticists attempting to solve physiological or pathological problems by means of candidate gene or genome-wide studies have met with little success, albeit with a few remarkable exceptions, such as the *BCL11A* modifier gene underlying the clinical presentation of beta hemoglobin disorders at population level (114), common variants at the type III IFN locus affecting both the spontaneous clearance of hepatitis C and the response to type I IFN therapy in association studies (115, 116, 117, 118), and the high risk of Crohn’s disease in homozygotes for *NOD2*-null alleles in linkage studies (119, 120). In our view, large population-based studies are more suitable for use in evolutionary than in physiological biology.

Galton’s misconception in his attempt to discover the laws of inheritance was perhaps related to the “tacit knowledge” that populations have genes (121). They don’t. Cells, tissues, organs, and organisms have genes. A group of organisms does not form a meta- or supra-organism. Genes do not operate, physiologically or pathologically, in populations. They operate in individuals. Both physiology and pathology are therefore best studied in organisms and families, not populations. This may be why Mendel cracked the laws of inheritance: he studied individual pea pods and their offspring, not randomly assembled populations of peas.

## Of genetics and immunology

In this light, we see “rare” versus “common” conditions as a false dichotomy (122). All human beings are unique, not only in terms of their position but, more importantly, in terms of their composition. This notion stems back to Claude Bernard (109). Diseases are “words.” The only reality is the patient. Thus, human genetics should serve physiological and pathological endeavors in individual patients and families. Each patient should be studied individually, deciphering a molecular chain of causes and consequences between genotype and phenotype (66).

The extraordinary genetic heterogeneity of humans was obvious to astute observers of human phenotypes, from Garrod onward, decades before its confirmation by genome sequencing. How could an almost unlimited phenotypic heterogeneity not be due to an even greater genetic heterogeneity? This diversity has contributed to the spectacular success of “vertical,” family-based genetics in human medicine and the relatively disappointing results for “horizontal,” population-based genetic studies performed for the same purpose (123).

Consistently, we are mindful that pediatrics is the gate to medicine and that genetics is its key. The genetic study of young patients is more likely to be fruitful. It can reveal a general physiological mechanism that may be disrupted in older patients by other, more common, causes. This notion has been neatly illustrated by the studies of tuberculosis and COVID-19, which were built on the studies of Mendelian susceptibility to mycobacterial disease and influenza (124).

We are also aware that the somatic genetic nature of adaptive immunity, with the creation of a gigantic repertoire of B and T cell receptors, which developed twice during the evolution of vertebrates by convergent evolution, attests to the need for evolution to mitigate the rigidity and insufficiency of the germline (97). We are well aware that this history explains the somatic, as opposed to germline, inclination of most immunological schools.

Nevertheless, genetics has driven at least three most extraordinary immunological achievements. MHC-based alloreactivity (125, 126), MHC restriction (127), and microbial sensors (128) were all discovered by unbiased genetic studies in mice for the last three studies and in humans for the first. Forward genetic studies of infection in mice have also been most successful, from the discoveries of MX and Nramp1 onward (129, 130). With modern tools, the potential of human genetics for studying immunity is even greater. There is little doubt that human genetics will play an increasing role in immunology, as in biology and medicine in general.

An extraordinary resource is around us, with billions of children, adults, and elderly people in need of a molecular and cellular explanation for their immunological illness. The prerequisites for the exploration of this uncharted terrain are now to hand: a medical team, genome sequencing, and a scientific team. It is clear to anyone whose vision is not obscured by the lens of habit that studying human immunity via the dissection of inborn errors is destined for a glorious future.

## Conclusion

What do we intend to publish? A journal publishes what its editors like. It is a matter of taste. We can only hope that the readers will appreciate our taste, which is based on the conviction that the determinism of human life operates in ever-changing, unique individuals and that inborn errors of immunity offer a unique opportunity to rethink and rewrite immunology (131) while offering patients and their families new life-saving opportunities.

The scope of this journal will include immunological and clinical studies based on genotypes that have a strong, causal impact on human phenotypes and their phenocopies. We are interested in reporting causes and consequences that operate in individual human beings, based on the discovery of inborn errors of immunity, bridging the gap between genotypes and phenotypes by means of in-depth molecular and cellular mechanistic studies. We will also consider studies of modifiers operating at population level if their impact is firmly established. Finally, we will welcome non-genetic immunological studies that pave the way for genetic studies (132, 133, 134, 135).

The study of causes and consequences can be conducted in any individual at any moment. This would, obviously, be logistically impossible for all individuals. However, we cannot reasonably hope to understand living organisms as we understand inert matter. Nominalism is inherent to biology, as typology is inherent to physics. We thus take pride in the publication of case reports, clinical series, and related papers.

**Figure 1.**
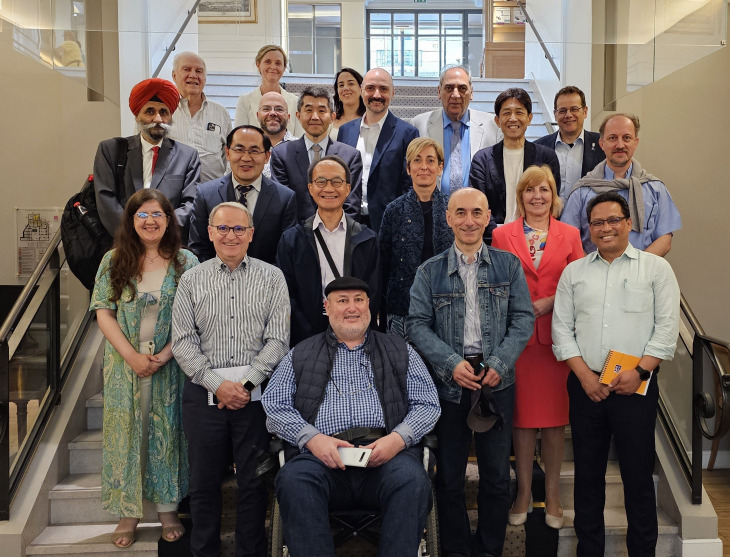
**This photo was taken in June 2023 in Paris.** Jean-Laurent Casanova (Editor of the *Journal of Clinical Immunology*) was hosting a reunion that included Vinny Bonagura (Editor of the *Journal of Clinical Immunology*), Sudhir Gupta (Founding Editor of the *Journal of Clinical Immunology*), Fabio Candotti (representing the European Society for Immunodeficiencies [ESID]), Elonora Gambineri (ESID), Stuart Tangye (Australasian Society of Clinical Immunology and Allergy [ASCIA]), Tomohiro Morio (Japanese Society for Immunodeficiency and Autoinflammatory Diseases [JSIAD]), Satoshi Okada (JSIAD), Surjit Singh (Asia-Pacific Society for Immunodeficiencies [APSID]), Yu-Lung Lau (APSID), Davood Mansoori (Iranian Primary Immunodeficiency Network [IPIN]), Nima Rezaei (IPIN), Biman Saikia (Indian Society for Primary Immune Deficiency [ISPID]), Elisaveta Naumova (J Project), Jinqiao Sun (Chinese Society for Inborn Errors of Immunity [CSIEI]), Dusan Bogunovic (Henry Kunkel Society [HKS]), Isabelle Meyts (International Union of Immunological Societies [IUIS], Committee for Inborn Errors of Immunity), Cecilia Poli (Latin American Society for Immunodeficiencies [LASID]), Aziz Bousfiha (Arab Society for Primary Immunodeficiencies [ARAPID]), Leila Jeddane (African Society for Immunodeficiencies [ASID]), and Elie Haddad (Clinical Immunology Society [CIS]). A representative of each of these 14 societies in the field of human inborn errors of immunity serves on the Board of Society Editors of the *Journal of Human Immunity*.

I will close this inaugural editorial by considering the circumstances that led to the launch of this new journal. It all started with a meeting in Paris in June 2023 with representatives from 14 major societies in the field of human inborn errors of immunity, including the five founding societies of the International Alliance of Primary Immunodeficiency Societies (IAPIDS) (Fig. 1). The societies and the editors of the *Journal of Clinical Immunology* (*JoCI*) were equally unhappy with the way Springer envisaged the future of *JoCI*. The editorial team of the *JoCI* resigned. The IAPIDS and Rockefeller University Press (RUP) joined forces in a joint venture, providing an alternative route and graciously inviting them to launch this new journal.

Our editors reflect this international effort. Megan Cooper has kindly agreed to be the Deputy Editor, while Dusan Bogunovic, Petter Brodin, Andy Gennery, Elena Hsieh, Isabelle Meyts, Tomohiro Morio, Cecilia Poli, Anne Puel, Neil Romberg, Vijay Sankaran, Helen Su, Stuart Tangye, Stuart Turvey, and Shen-Ying Zhang have agreed to become involved as Associate Editors and Yanick Crow, Josh Milner, and Luigi Notarangelo have agreed to be Consulting Editors. We also have a prestigious Scientific Advisory Board, including 22 members of the National Academy of Sciences of the USA. Finally, we have a Board of Regional Editors, with members from almost all nations and most regions of large countries, and a Board of Society Editors, with representatives from each of the 14 societies that attended the Paris meeting.

We now invite all colleagues in the field of human inborn errors of immunity to submit their best papers to the *Journal of Human Immunity*. We also encourage teams studying human immunity that are not familiar with human genetics to browse this journal and to think on how they too could conduct similar studies. They should not hesitate to contact the many teams in the field, which they will find to be warm, welcoming, and inclusive. We see this journal as a medium for allowing our field to continue to grow beyond its current limits.

## References

[1] Garrod, A.E. 1931. The Inborn Factors in Disease: An Essay. Clarendon Press, Oxford, UK. 10.1097/00007611-193106000-00028

[2] Bearn, A.G. 1993. Archibald Garrod and the Individuality of Man. Clarendon Press, Oxford, UK.

[3] Beadle, G.W. 1974. Recollections. Annu. Rev. Biochem. 43:1–13. 10.1146/annurev.bi.43.070174.000245 4605017

[4] Pauling, L., H.A. Itano, S.J. Singer, and I.C. Wells. 1949. Sickle cell anemia a molecular disease. Science. 110:543–548. 10.1126/science.110.2865.543 15395398

[5] Ingram, V.M. 1956. A specific chemical difference between the globins of normal human and sickle-cell anaemia haemoglobin. Nature. 178:792–794. 10.1038/178792a0 13369537

[6] Mayr, E. 1988. Toward a New Philosophy of Biology: Observations of an Evolutionist. Harvard University Press, Cambridge, MA, USA.

[7] Poli, M.C., I. Aksentijevich, A. Bousfiha, C. Cunningham-Rundles, S. Hambleton, C. Klein, T. Morio, C. Picard, A. Puel, N. Rezaei, . 2025. Human inborn errors of immunity: 2024 update on the classification from the International Union of Immunological Societies Expert Committee. J. Hum. Immun. In press. 10.70962/jhi.20250003 PMC12829761 41608114

[8] Bousfiha, A., L. Jeddane, A. Moundir, M.C. Poli, I. Aksentijevich, C. Cunningham-Rundles, S. Hambleton, C. Klein, T. Morio, C. Picard, . 2025. The 2024 update of IUIS phenotypical classification for human inborn errors of immunity. J. Hum. Immun. In press. 10.70962/jhi.20250002 PMC12829316 41608113

[9] Bonthron, D.T., A.F. Markham, D. Ginsburg, and S.H. Orkin. 1985. Identification of a point mutation in the adenosine deaminase gene responsible for immunodeficiency. J. Clin. Invest. 76:894–897. 10.1172/JCI112050 3839802 PMC423929

[10] Notarangelo, L.D., R. Bacchetta, J.L. Casanova, and H.C. Su. 2020. Human inborn errors of immunity: An expanding universe. Sci. Immunol. 5:eabb1662. 10.1126/sciimmunol.abb1662 32651211 PMC7647049

[11] Akalu, Y.T., and D. Bogunovic. 2024. Inborn errors of immunity: An expanding universe of disease and genetic architecture. Nat. Rev. Genet. 25:184–195. 10.1038/s41576-023-00656-z 37863939 PMC13489759

[12] Lucas, C.L. 2024. Human genetic errors of immunity illuminate an adaptive arsenal model of rapid defenses. Trends Immunol. 45:113–126. 10.1016/j.it.2023.12.006 38302340 PMC12442919

[13] Suzuki, T., T. Sakagami, B.K. Rubin, L.M. Nogee, R.E. Wood, S.L. Zimmerman, T. Smolarek, M.K. Dishop, S.E. Wert, J.A. Whitsett, . 2008. Familial pulmonary alveolar proteinosis caused by mutations in CSF2RA. J. Exp. Med. 205:2703–2710. 10.1084/jem.20080990 18955570 PMC2585845

[14] IRF4 International Consortium, Fornes, O., A. Jia, H.S. Kuehn, Q. Min, U. Pannicke, N. Schleussner, R. Thouenon, Z. Yu, M. de Los Angeles Astbury, . 2023. A multimorphic mutation in IRF4 causes human autosomal dominant combined immunodeficiency. Sci. Immunol. 8:eade7953. 10.1126/sciimmunol.ade7953 36662884 PMC10825898

[15] Yamashita, M., H.S. Kuehn, K. Okuyama, S. Okada, Y. Inoue, N. Mitsuiki, K. Imai, M. Takagi, H. Kanegane, M. Takeuchi, . 2021. A variant in human AIOLOS impairs adaptive immunity by interfering with IKAROS. Nat. Immunol. 22:893–903. 10.1038/s41590-021-00951-z 34155405 PMC8958960

[16] Mizoguchi, Y., and S. Okada. 2021. Inborn errors of STAT1 immunity. Curr. Opin. Immunol. 72:59–64. 10.1016/j.coi.2021.02.009 33839590

[17] Bosticardo, M., K. Dobbs, O.M. Delmonte, A.J. Martins, F. Pala, T. Kawai, H. Kenney, G. Magro, L.B. Rosen, Y. Yamazaki, . 2025. Multiomics dissection of human RAG deficiency reveals distinctive patterns of immune dysregulation but a common inflammatory signature. Sci Immunol. 10:eadq1697. 10.1126/sciimmunol.adq1697 39792639 PMC12087669

[18] Gruber, C., and D. Bogunovic. 2020. Incomplete penetrance in primary immunodeficiency: A skeleton in the closet. Hum. Genet. 139:745–757. 10.1007/s00439-020-02131-9 32067110 PMC7275875

[19] Stewart, O., C. Gruber, H.E. Randolph, R. Patel, M. Ramba, E. Calzoni, L.H. Huang, J. Levy, S. Buta, A. Lee, . 2025. Monoallelic expression can govern penetrance of inborn errors of immunity. Nature. 637:1186–1197. 10.1038/s41586-024-08346-4 39743591 PMC11804961

[20] Israel, L., Y. Wang, K. Bulek, E. Della Mina, Z. Zhang, V. Pedergnana, M. Chrabieh, N.A. Lemmens, V. Sancho-Shimizu, M. Descatoire, . 2017. Human adaptive immunity rescues an inborn error of innate immunity. Cell. 168:789–800.e10. 10.1016/j.cell.2017.01.039 28235196 PMC5328639

[21] Spaan, A.N., A.L. Neehus, E. Laplantine, F. Staels, M. Ogishi, Y. Seeleuthner, F. Rapaport, K.A. Lacey, E. Van Nieuwenhove, M. Chrabieh, . 2022. Human OTULIN haploinsufficiency impairs cell-intrinsic immunity to staphylococcal α-toxin. Science. 376:eabm6380. 10.1126/science.abm6380 35587511 PMC9233084

[22] Casanova, J.L., and L. Abel. 2018. Human genetics of infectious diseases: Unique insights into immunological redundancy. Semin. Immunol. 36:1–12. 10.1016/j.smim.2017.12.008 29254755 PMC5910248

[23] French FMF Consortium . 1997. A candidate gene for familial Mediterranean fever. Nat. Genet. 17:25–31. 10.1038/ng0997-25 9288094

[24] The International FMF Consortium . 1997. Ancient missense mutations in a new member of the RoRet gene family are likely to cause familial Mediterranean fever. Cell. 90:797–807. 10.1016/S0092-8674(00)80539-5 9288758

[25] Boisson-Dupuis, S., N. Ramirez-Alejo, Z. Li, E. Patin, G. Rao, G. Kerner, C.K. Lim, D.N. Krementsov, N. Hernandez, C.S. Ma, . 2018. Tuberculosis and impaired IL-23-dependent IFN-γ immunity in humans homozygous for a common TYK2 missense variant. Sci. Immunol. 3:eaau8714. 10.1126/sciimmunol.aau8714 30578352 PMC6341984

[26] Bastard, P., K.C. Hsiao, Q. Zhang, J. Choin, E. Best, J. Chen, A. Gervais, L. Bizien, M. Materna, C. Harmant, . 2022. A loss-of-function IFNAR1 allele in Polynesia underlies severe viral diseases in homozygotes. J. Exp. Med. 219:e20220028. 10.1084/jem.20220028 35442418 PMC9026234

[27] Duncan, C.J.A., M.K. Skouboe, S. Howarth, A.K. Hollensen, R. Chen, M.L. Børresen, B.J. Thompson, J. Stremenova Spegarova, C.F. Hatton, F.F. Stæger, . 2022. Life-threatening viral disease in a novel form of autosomal recessive IFNAR2 deficiency in the Arctic. J. Exp. Med. 219:e20212427. 10.1084/jem.20212427 35442417 PMC9026249

[28] Meyts, I. 2022. Null IFNAR1 and IFNAR2 alleles are surprisingly common in the Pacific and Arctic. J. Exp. Med. 219:e20220491. 10.1084/jem.20220491 35486090 PMC9070088

[29] Aluri, J., and M.A. Cooper. 2023. Somatic mosaicism in inborn errors of immunity: Current knowledge, challenges, and future perspectives. Semin. Immunol. 67:101761. 10.1016/j.smim.2023.101761 37062181 PMC11321052

[30] Casanova, J.L., J. Peel, J. Donadieu, A.L. Neehus, A. Puel, and P. Bastard. 2024. The ouroboros of autoimmunity. Nat. Immunol. 25:743–754. 10.1038/s41590-024-01815-y 38698239

[31] Goldschmidt, R.B. 1945. Additional data on phenocopies and genic action. J. Exp. Zool. 100:193–201. 10.1002/jez.1401000203 21003382

[32] Beck, D.B., M.A. Ferrada, K.A. Sikora, A.K. Ombrello, J.C. Collins, W. Pei, N. Balanda, D.L. Ross, D. Ospina Cardona, Z. Wu, . 2020. Somatic mutations in UBA1 and severe adult-onset autoinflammatory disease. N. Engl. J. Med. 383:2628–2638. 10.1056/NEJMoa2026834 33108101 PMC7847551

[33] Goodnow, C.C. 2007. Multistep pathogenesis of autoimmune disease. Cell. 130:25–35. 10.1016/j.cell.2007.06.033 17632054

[34] Bruton, O.C. 1952. Agammaglobulinemia. Pediatrics. 9:722–728. 10.1542/peds.9.6.722 14929630

[35] Lutz, W. 1946. [About verruciform epidermodysplasia]. Dermatologica. 92:30–43. 10.1159/000255805 20982046

[36] Tiri, A., R. Masetti, F. Conti, A. Tignanelli, E. Turrini, P. Bertolini, S. Esposito, and A. Pession. 2021. Inborn errors of immunity and cancer. Biology (Basel). 10:313. 10.3390/biology10040313 33918597 PMC8069273

[37] Casanova, J.L., and L. Abel. 2024. The Microbe, the infection enigma, and the host. Annu. Rev. Microbiol. 78:103–124. 10.1146/annurev-micro-092123-022855 38986133 PMC11956784

[38] Casanova, J.L., and L. Abel. 2020. The human genetic determinism of life-threatening infectious diseases: Genetic heterogeneity and physiological homogeneity? Hum. Genet. 139:681–694. 10.1007/s00439-020-02184-w 32462426 PMC7251220

[39] Agnello, V., M.M. De Bracco, and H.G. Kunkel. 1972. Hereditary C_2_ deficiency with some manifestations of systemic lupus erythematosus. J. Immunol. 108:837–840. 10.4049/jimmunol.108.3.837 4110990

[40] Moncada, B., N.K. Day, R.A. Good, and D.B. Windhorst. 1972. Lupus-erythematosus-like syndrome with a familial defect of complement. N. Engl. J. Med. 286:689–693. 10.1056/NEJM197203302861304 4110615

[41] Uggenti, C., A. Lepelley, and Y.J. Crow. 2019. Self-awareness: Nucleic acid-driven inflammation and the type I interferonopathies. Annu. Rev. Immunol. 37:247–267. 10.1146/annurev-immunol-042718-041257 30633609

[42] Masters, S.L., A. Simon, I. Aksentijevich, and D.L. Kastner. 2009. Horror autoinflammaticus: The molecular pathophysiology of autoinflammatory disease (*). Annu. Rev. Immunol. 27:621–668. 10.1146/annurev.immunol.25.022106.141627 19302049 PMC2996236

[43] Donaldson, V.H., and R.R. Evans. 1963. A biochemical abnormality in herediatry angioneurotic edema: Absence of serum inhibitor of C′ 1-Esterase. Am. J. Med. 35:37–44. 10.1016/0002-9343(63)90162-1 14046003

[44] Sharma, M., D. Leung, M. Momenilandi, L.C.W. Jones, L. Pacillo, A.E. James, J.R. Murrell, S. Delafontaine, J. Maimaris, M. Vaseghi-Shanjani, . 2023. Human germline heterozygous gain-of-function STAT6 variants cause severe allergic disease. J. Exp. Med. 220:e20221755. 10.1084/jem.20221755 36884218 PMC10037107

[45] Arkwright, P.D., and A.R. Gennery. 2011. Ten warning signs of primary immunodeficiency: A new paradigm is needed for the 21st century. Ann. N. Y. Acad. Sci. 1238:7–14. 10.1111/j.1749-6632.2011.06206.x 22129048

[46] Casanova, J.L., and L. Abel. 2007. Primary immunodeficiencies: A field in its infancy. Science. 317:617–619. 10.1126/science.1142963 17673650

[47] Ochs, H.D., C.I.E. Smith, and J. Puck. 2014. Primary Immunodeficiency Diseases: A Molecular and Genetic Approach. Third edition. Oxford University Press, Oxford, UK. 10.1093/med/9780195389838.001.0001

[48] Tsukada, S., D.C. Saffran, D.J. Rawlings, O. Parolini, R.C. Allen, I. Klisak, R.S. Sparkes, H. Kubagawa, T. Mohandas, S. Quan, . 1993. Deficient expression of a B cell cytoplasmic tyrosine kinase in human X-linked agammaglobulinemia. Cell. 72:279–290. 10.1016/0092-8674(93)90667-F 8425221

[49] Nagamine, K., P. Peterson, H.S. Scott, J. Kudoh, S. Minoshima, M. Heino, K.J. Krohn, M.D. Lalioti, P.E. Mullis, S.E. Antonarakis, . 1997. Positional cloning of the APECED gene. Nat. Genet. 17:393–398. 10.1038/ng1297-393 9398839

[50] Finnish-German APECED Consortium . 1997. An autoimmune disease, APECED, caused by mutations in a novel gene featuring two PHD-type zinc-finger domains. Nat. Genet. 17:399–403. 10.1038/ng1297-399 9398840

[51] Husebye, E.S., M.S. Anderson, and O. Kämpe. 2018. Autoimmune polyendocrine syndromes. N. Engl. J. Med. 378:2543–2544. 10.1056/NEJMra1713301 29949487

[52] Tangye, S.G., T. Nguyen, E.K. Deenick, V.L. Bryant, and C.S. Ma. 2023. Inborn errors of human B cell development, differentiation, and function. J. Exp. Med. 220:e20221105. 10.1084/jem.20221105 37273190 PMC10242086

[53] Le Coz, C., D.N. Nguyen, C. Su, B.E. Nolan, A.V. Albrecht, S. Xhani, D. Sun, B. Demaree, P. Pillarisetti, C. Khanna, . 2021. Constrained chromatin accessibility in PU.1-mutated agammaglobulinemia patients. J. Exp. Med. 218:e20201750. 10.1084/jem.20201750 33951726 PMC8105723

[54] Royer-Pokora, B., L.M. Kunkel, A.P. Monaco, S.C. Goff, P.E. Newburger, R.L. Baehner, F.S. Cole, J.T. Curnutte, and S.H. Orkin. 1986. Cloning the gene for an inherited human disorder–chronic granulomatous disease–on the basis of its chromosomal location. Nature. 322:32–38. 10.1038/322032a0 2425263

[55] Teahan, C., P. Rowe, P. Parker, N. Totty, and A.W. Segal. 1987. The X-linked chronic granulomatous disease gene codes for the beta-chain of cytochrome b-245. Nature. 327:720–721. 10.1038/327720a0 3600769

[56] Crow, Y.J., and J.L. Casanova. 2024. Human life within a narrow range: The lethal ups and downs of type I interferons. Sci. Immunol. 9:eadm8185. 10.1126/sciimmunol.adm8185 38968338

[57] Ridanpää, M., H. van Eenennaam, K. Pelin, R. Chadwick, C. Johnson, B. Yuan, W. vanVenrooij, G. Pruijn, R. Salmela, S. Rockas, . 2001. Mutations in the RNA component of RNase MRP cause a pleiotropic human disease, cartilage-hair hypoplasia. Cell. 104:195–203. 10.1016/S0092-8674(01)00205-7 11207361

[58] Xu, G.L., T.H. Bestor, D. Bourc’his, C.L. Hsieh, N. Tommerup, M. Bugge, M. Hulten, X. Qu, J.J. Russo, and E. Viegas-Péquignot. 1999. Chromosome instability and immunodeficiency syndrome caused by mutations in a DNA methyltransferase gene. Nature. 402:187–191. 10.1038/46052 10647011

[59] Bruton, O.C. 1962. A decade with agammaglobulinemia. J. Pediatr. 60:672–676. 10.1016/S0022-3476(62)80092-4 13874104

[60] Gatti, R.A., H.J. Meuwissen, H.D. Allen, R. Hong, and R.A. Good. 1968. Immunological reconstitution of sex-linked lymphopenic immunological deficiency. Lancet. 2:1366–1369. 10.1016/S0140-6736(68)92673-1 4177932

[61] Polmar, S.H., R.C. Stern, A.L. Schwartz, E.M. Wetzler, P.A. Chase, and R. Hirschhorn. 1976. Enzyme replacement therapy for adenosine deaminase deficiency and severe combined immunodeficiency. N. Engl. J. Med. 295:1337–1343. 10.1056/NEJM197612092952402 980079

[62] Hirschhorn, R., D.R. Yang, J.M. Puck, M.L. Huie, C.K. Jiang, and L.E. Kurlandsky. 1996. Spontaneous in vivo reversion to normal of an inherited mutation in a patient with adenosine deaminase deficiency. Nat. Genet. 13:290–295. 10.1038/ng0796-290 8673127

[63] Cavazzana-Calvo, M., S. Hacein-Bey, G. de Saint Basile, F. Gross, E. Yvon, P. Nusbaum, F. Selz, C. Hue, S. Certain, J.L. Casanova, . 2000. Gene therapy of human severe combined immunodeficiency (SCID)-X1 disease. Science. 288:669–672. 10.1126/science.288.5466.669 10784449

[64] McAuley, G.E., G. Yiu, P.C. Chang, G.A. Newby, B. Campo-Fernandez, S.T. Fitz-Gibbon, X. Wu, S.L. Kang, A. Garibay, J. Butler, . 2023. Human T cell generation is restored in CD3δ severe combined immunodeficiency through adenine base editing. Cell. 186:1398–1416.e23. 10.1016/j.cell.2023.02.027 36944331 PMC10876291

[65] Thakar, M.S., B.R. Logan, J.M. Puck, E.A. Dunn, R.H. Buckley, M.J. Cowan, R.J. O’Reilly, N. Kapoor, L.F. Satter, S.Y. Pai, . 2023. Measuring the effect of newborn screening on survival after haematopoietic cell transplantation for severe combined immunodeficiency: A 36-year longitudinal study from the primary immune deficiency treatment consortium. Lancet. 402:129–140. 10.1016/S0140-6736(23)00731-6 37352885 PMC10386791

[66] Casanova, J.L., M.E. Conley, S.J. Seligman, L. Abel, and L.D. Notarangelo. 2014. Guidelines for genetic studies in single patients: Lessons from primary immunodeficiencies. J. Exp. Med. 211:2137–2149. 10.1084/jem.20140520 25311508 PMC4203950

[67] Lu, W., Y. Zhang, D.O. McDonald, H. Jing, B. Carroll, N. Robertson, Q. Zhang, H. Griffin, S. Sanderson, J.H. Lakey, . 2014. Dual proteolytic pathways govern glycolysis and immune competence. Cell. 159:1578–1590. 10.1016/j.cell.2014.12.001 25525876 PMC4297473

[68] Ham, H., H. Jing, I.T. Lamborn, M.M. Kober, A. Koval, Y.A. Berchiche, D.E. Anderson, K.M. Druey, J.N. Mandl, B. Isidor, . 2024. Germline mutations in a G protein identify signaling cross-talk in T cells. Science. 385:eadd8947. 10.1126/science.add8947 39298586 PMC11811912

[69] Zhang, S.Y., N.E. Clark, C.A. Freije, E. Pauwels, A.J. Taggart, S. Okada, H. Mandel, P. Garcia, M.J. Ciancanelli, A. Biran, . 2018. Inborn errors of RNA Lariat metabolism in humans with brainstem viral infection. Cell. 172:952–965.e18. 10.1016/j.cell.2018.02.019 29474921 PMC5886375

[70] Bohlen, J., Q. Zhou, Q. Philippot, M. Ogishi, D. Rinchai, T. Nieminen, S. Seyedpour, N. Parvaneh, N. Rezaei, N. Yazdanpanah, . 2023. Human MCTS1-dependent translation of JAK2 is essential for IFN-γ immunity to mycobacteria. Cell. 186:5114–5134.e27. 10.1016/j.cell.2023.09.024 37875108 PMC10841658

[71] Rosen, F.S., R.J. Wedgwood, F. Aiuti, M.D. Cooper, R.A. Good, L.A. Hanson, W.H. Hitzig, S. Matsumoto, M. Seligmann, J.F. Soothill, and T.A. Waldmann. 1983. Primary immunodeficiency diseases: Report prepared for the WHO by a scientific group on immunodeficiency. Clin. Immunol. Immunopathol. 28:450–475. 10.1016/0090-1229(83)90112-5 6349887

[72] Bastard, P., L.B. Rosen, Q. Zhang, E. Michailidis, H.H. Hoffmann, Y. Zhang, K. Dorgham, Q. Philippot, J. Rosain, V. Béziat, . 2020. Autoantibodies against type I IFNs in patients with life-threatening COVID-19. Science. 370:eabd4585. 10.1126/science.abd4585 32972996 PMC7857397

[73] Bastard, P., A. Gervais, T. Le Voyer, J. Rosain, Q. Philippot, J. Manry, E. Michailidis, H.H. Hoffmann, S. Eto, M. Garcia-Prat, . 2021. Autoantibodies neutralizing type I IFNs are present in ∼4% of uninfected individuals over 70 years old and account for ∼20% of COVID-19 deaths. Sci. Immunol. 6:eabl4340. 10.1126/sciimmunol.abl4340 34413139 PMC8521484

[74] Gervais, A., F. Rovida, M.A. Avanzini, S. Croce, A. Marchal, S.C. Lin, A. Ferrari, C.W. Thorball, O. Constant, T. Le Voyer, . 2023. Autoantibodies neutralizing type I IFNs underlie West Nile virus encephalitis in ∼40% of patients. J. Exp. Med. 220:e20230661. 10.1084/jem.20230661 37347462 PMC10287549

[75] Kindt, T.J., and J.D. Capra. 1984. The Antibody Enigma. First edition. Plenum Press, New York, NY, USA. 10.1007/978-1-4684-4676-0

[76] Zhang, S.Y., and J.L. Casanova. 2024. Genetic defects of brain immunity in childhood herpes simplex encephalitis. Nature. 635:563–573. 10.1038/s41586-024-08119-z 39567785 PMC11822754

[77] Alper, C.A., A.M. Johnson, A.G. Birtch, and F.D. Moore. 1969. Human C’3: Evidence for the liver as the primary site of synthesis. Science. 163:286–288. 10.1126/science.163.3864.286 4883617

[78] Gresser, I. 1997. Wherefore interferon? J. Leukoc. Biol. 61:567–574. 10.1002/jlb.61.5.567 9129205

[79] Yan, N., and Z.J. Chen. 2012. Intrinsic antiviral immunity. Nat. Immunol. 13:214–222. 10.1038/ni.2229 22344284 PMC3549670

[80] Randow, F., J.D. MacMicking, and L.C. James. 2013. Cellular self-defense: How cell-autonomous immunity protects against pathogens. Science. 340:701–706. 10.1126/science.1233028 23661752 PMC3863583

[81] Bieniasz, P.D. 2004. Intrinsic immunity: A front-line defense against viral attack. Nat. Immunol. 5:1109–1115. 10.1038/ni1125 15496950

[82] Paludan, S.R., T. Pradeu, S.L. Masters, and T.H. Mogensen. 2021. Constitutive immune mechanisms: Mediators of host defence and immune regulation. Nat. Rev. Immunol. 21:137–150. 10.1038/s41577-020-0391-5 32782357 PMC7418297

[83] Kuhn, T. 1962. The Structure of Scientific Revolutions. University of Chicago Press, Chicago, IL, USA.

[84] Polanyi, M. 1958. Personal Knowledge: Towards a Post-Critical Philosophy. University of Chicago Press, Chicago, IL, USA.

[85] Timmins, A. 2013. Why was Kuhn’s structure more successful than Polanyi’s personal knowledge? J. Int. Soc. Hist. Philos. Sci. 3:306–317. 10.1086/671347

[86] Guillemin, R. 2011. Neuroendocrinology: A short historical review. Ann. N. Y. Acad. Sci. 1220:1–5. 10.1111/j.1749-6632.2010.05936.x 21388398

[87] Bearn, A.G., F.J. Dixon, and B. Benacerraf. 1985. Henry G. Kunkel 1916-1983. An appreciation of the man and his scientific contributions & a bibliography of his research papers. J. Exp. Med. 161:869–895. 10.1084/jem.161.5.869 3886830 PMC2187592

[88] Medetgul-Ernar, K., and M.M. Davis. 2022. Standing on the shoulders of mice. Immunity. 55:1343–1353. 10.1016/j.immuni.2022.07.008 35947979 PMC10035762

[89] Casanova, J.L., and L. Abel. 2002. Genetic dissection of immunity to mycobacteria: The human model. Annu. Rev. Immunol. 20:581–620. 10.1146/annurev.immunol.20.081501.125851 11861613

[90] Casanova, J.L., and L. Abel. 2004. The human model: A genetic dissection of immunity to infection in natural conditions. Nat. Rev. Immunol. 4:55–66. 10.1038/nri1264 14704768

[91] Rowe, R.G., and G.Q. Daley. 2019. Induced pluripotent stem cells in disease modelling and drug discovery. Nat. Rev. Genet. 20:377–388. 10.1038/s41576-019-0100-z 30737492 PMC6584039

[92] Farber, D.L. 2021. Tissues, not blood, are where immune cells function. Nature. 593:506–509. 10.1038/d41586-021-01396-y 34035530

[93] Gros, P., and J.L. Casanova. 2023. Reconciling mouse and human immunology at the altar of genetics. Annu. Rev. Immunol. 41:39–71. 10.1146/annurev-immunol-101721-065201 36525691

[94] Pradel, E., and J.J. Ewbank. 2004. Genetic models in pathogenesis. Annu. Rev. Genet. 38:347–363. 10.1146/annurev.genet.38.072902.092528 15568980

[95] Lemaitre, B., and J. Hoffmann. 2007. The host defense of Drosophila melanogaster. Annu. Rev. Immunol. 25:697–743. 10.1146/annurev.immunol.25.022106.141615 17201680

[96] Dangl, J.L., and J.D.G. Jones. 2024. A common immune response node in diverse plants. Science. 386:1344–1346. 10.1126/science.adu4930 39700299

[97] Boehm, T., M. Hirano, S.J. Holland, S. Das, M. Schorpp, and M.D. Cooper. 2018. Evolution of alternative adaptive immune systems in vertebrates. Annu. Rev. Immunol. 36:19–42. 10.1146/annurev-immunol-042617-053028 29144837

[98] Kostmann, R. 1950. Hereditär reticulos-en ny systemsjukdom. Sven. Laekartidn. 47:2861–2868.

[99] Hitzig, W.H., Z. Biro, H. Bosch, and H.J. Huser. 1958. [Agammaglobulinemia & alymphocytosis with atrophy of lymphatic tissue]. Helv. Paediatr. Acta. 13:551–585. 13640454

[100] Good, R.A. 1976. Presidential address to the American Association of Immunologists, delivered in Anaheim, California, April 13, 1976. Runestones in immunology: Inscriptions to journeys of discovery and analysis. J. Immunol. 117:1413–1428. 10.4049/jimmunol.117.5_Part_1.1413 794408

[101] Cooper, M.D. 2003. In memoriam. Robert A. Good May 21, 1922-June 13, 2003. J. Immunol. 171:6318–6319. 10.4049/jimmunol.171.12.6318 14662826

[102] Miller, J.F. 2004. Events that led to the discovery of T-cell development and function–a personal recollection. Tissue Antigens. 63:509–517. 10.1111/j.0001-2815.2004.00255.x 15140026

[103] Cooper, M.D., R.D. Peterson, and R.A. Good. 1965. A new concept of the cellular basis of immunity. J. Pediatr. 67:907–908. 10.1016/S0022-3476(65)81796-6

[104] Etzioni, A., and H.D. Ochs. 2014. Primary Immunodeficiency Disorders: A Historic and Scientific Perspective. First edition. Elsevier Science & Technology, Chantilly, VA, USA. 10.1016/C2012-0-03311-X

[105] McQuarrie, I. 1944. The Experiments of Nature, and Other Essays. University extension division, University of Kansas, Lawrence, KS, USA.

[106] Garrod, A.E. 1926. Science of clinical medicine. Lancet. 208:735–737. 10.1016/S0140-6736(01)26654-9

[107] Good, R.A. 2024. “The Minnesota Scene: A Crucial Portal of Entry to Modern Cellular Immunology” in the Immunologic Revolution. CRC Press, Boca Raton, FL, USA. 105–168.

[108] Krogh, A. 1929. The Progress of Physiology. Science. 70:200–204. 10.1126/science.70.1809.200 17732865

[109] Bernard, C. 1957. An Introduction to the Study of Experimental Medicine. Vol. 400. Courier Corporation, North Chelmsford, MA, USA.

[110] Jørgensen, C.B. 2001. August Krogh and Claude Bernard on basic principles in experimental physiology. Bioscience. 51:59–61. 10.1641/0006-3568(2001)051[0059:AKACBO]2.0.CO;2

[111] Brenner, S. 2001. My Life in Science. Biomed Central Limited, London, UK.

[112] Brenner, S. 2007. The human genome: The nature of the enterprise. *In* 1990 Human Genetic Information: Science, Law and Ethics. D.J. Chadwick, G. Bock, and J. Whelan, editors. Wiley, Chichester, UK. 6–17.

[113] Patin, E., and L. Quintana-Murci. 2024. Tracing the evolution of human immunity through Ancient DNA. Annu. Rev. Immunol. 10.1146/annurev-immunol-082323-024638 39705165

[114] Sankaran, V.G., T.F. Menne, J. Xu, T.E. Akie, G. Lettre, B. Van Handel, H.K. Mikkola, J.N. Hirschhorn, A.B. Cantor, and S.H. Orkin. 2008. Human fetal hemoglobin expression is regulated by the developmental stage-specific repressor BCL11A. Science. 322:1839–1842. 10.1126/science.1165409 19056937

[115] Ge, D., J. Fellay, A.J. Thompson, J.S. Simon, K.V. Shianna, T.J. Urban, E.L. Heinzen, P. Qiu, A.H. Bertelsen, A.J. Muir, . 2009. Genetic variation in IL28B predicts hepatitis C treatment-induced viral clearance. Nature. 461:399–401. 10.1038/nature08309 19684573

[116] Thomas, D.L., C.L. Thio, M.P. Martin, Y. Qi, D. Ge, C. O’Huigin, J. Kidd, K. Kidd, S.I. Khakoo, G. Alexander, . 2009. Genetic variation in IL28B and spontaneous clearance of hepatitis C virus. Nature. 461:798–801. 10.1038/nature08463 19759533 PMC3172006

[117] Suppiah, V., M. Moldovan, G. Ahlenstiel, T. Berg, M. Weltman, M.L. Abate, M. Bassendine, U. Spengler, G.J. Dore, E. Powell, . 2009. IL28B is associated with response to chronic hepatitis C interferon-alpha and ribavirin therapy. Nat. Genet. 41:1100–1104. 10.1038/ng.447 19749758

[118] Tanaka, Y., N. Nishida, M. Sugiyama, M. Kurosaki, K. Matsuura, N. Sakamoto, M. Nakagawa, M. Korenaga, K. Hino, S. Hige, . 2009. Genome-wide association of IL28B with response to pegylated interferon-alpha and ribavirin therapy for chronic hepatitis C. Nat. Genet. 41:1105–1109. 10.1038/ng.449 19749757

[119] Hugot, J.P., M. Chamaillard, H. Zouali, S. Lesage, J.P. Cézard, J. Belaiche, S. Almer, C. Tysk, C.A. O’Morain, M. Gassull, . 2001. Association of NOD2 leucine-rich repeat variants with susceptibility to Crohn’s disease. Nature. 411:599–603. 10.1038/35079107 11385576

[120] Ogura, Y., D.K. Bonen, N. Inohara, D.L. Nicolae, F.F. Chen, R. Ramos, H. Britton, T. Moran, R. Karaliuskas, R.H. Duerr, . 2001. A frameshift mutation in NOD2 associated with susceptibility to Crohn’s disease. Nature. 411:603–606. 10.1038/35079114 11385577

[121] Gillham, N.W. 2001. A Life of Sir Francis Galton: From African Exploration to the Birth of Eugenics. Oxford University Press, Oxford, UK. 10.1093/oso/9780195143652.001.0001

[122] Casanova, J.L., and M.S. Anderson. 2023. Unlocking life-threatening COVID-19 through two types of inborn errors of type I IFNs. J. Clin. Invest. 133:e166283. 10.1172/JCI166283 36719370 PMC9888384

[123] McClellan, J., and M.C. King. 2010. Genetic heterogeneity in human disease. Cell. 141:210–217. 10.1016/j.cell.2010.03.032 20403315

[124] Casanova, J.L., and L. Abel. 2022. From rare disorders of immunity to common determinants of infection: Following the mechanistic thread. Cell. 185:3086–3103. 10.1016/j.cell.2022.07.004 35985287 PMC9386946

[125] Dausset, J., and F.T. Rapaport. 1977. Immunology and genetics of transplantation. Perspect. Nephrol. Hypertens. 6:97–138. 138847

[126] Snell, G.D., M. Cherry, and P. Démant. 1973. H-2: Its structure and similarity to HL-A. Transpl. Rev. 15:3–25. 10.1111/j.1600-065X.1973.tb00108.x 4130131

[127] Doherty, P.C., and R.M. Zinkernagel. 1975. H-2 compatibility is required for T-cell-mediated lysis of target cells infected with lymphocytic choriomeningitis virus. J. Exp. Med. 141:502–507. 10.1084/jem.141.2.502 123002 PMC2190521

[128] Poltorak, A., X. He, I. Smirnova, M.Y. Liu, C. Van Huffel, X. Du, D. Birdwell, E. Alejos, M. Silva, C. Galanos, . 1998. Defective LPS signaling in C3H/HeJ and C57BL/10ScCr mice: Mutations in Tlr4 gene. Science. 282:2085–2088. 10.1126/science.282.5396.2085 9851930

[129] Staeheli, P., O. Haller, W. Boll, J. Lindenmann, and C. Weissmann. 1986. Mx protein: Constitutive expression in 3T3 cells transformed with cloned Mx cDNA confers selective resistance to influenza virus. Cell. 44:147–158. 10.1016/0092-8674(86)90493-9 3000619

[130] Vidal, S.M., D. Malo, K. Vogan, E. Skamene, and P. Gros. 1993. Natural resistance to infection with intracellular parasites: Isolation of a candidate for Bcg. Cell. 73:469–485. 10.1016/0092-8674(93)90135-D 8490962

[131] Nathan, C. 2021. Rethinking immunology. Science. 373:276–277. 10.1126/science.abj5637 34437138

[132] Pulendran, B., and M.M. Davis. 2020. The science and medicine of human immunology. Science. 369:eaay4014. 10.1126/science.aay4014 32973003 PMC7872131

[133] Brodin, P. 2022. Immune-microbe interactions early in life: A determinant of health and disease long term. Science. 376:945–950. 10.1126/science.abk2189 35617387

[134] Sallusto, F. 2016. Heterogeneity of Human CD4(+) T Cells Against Microbes. Annu. Rev. Immunol. 34:317–334. 10.1146/annurev-immunol-032414-112056 27168241

[135] Tan, J., L. Piccoli, and A. Lanzavecchia. 2019. The antibody response to Plasmodium falciparum: Cues for vaccine design and the discovery of receptor-based antibodies. Annu. Rev. Immunol. 37:225–246. 10.1146/annurev-immunol-042617-053301 30566366

